# An Adaptive Control Scheme Based on Non-Interference Nonlinearity Approximation for a Class of Nonlinear Cascaded Systems and Its Application to Flexible Joint Manipulators

**DOI:** 10.3390/s24103178

**Published:** 2024-05-16

**Authors:** Zhangxing Liu, Hongzhe Jin, Jie Zhao

**Affiliations:** School of Mechatronics Engineering, Harbin Institute of Technology, Harbin 150001, China; liuzhangxinghit@163.com (Z.L.); jzhao@hit.edu.cn (J.Z.)

**Keywords:** model-free adaptive control, nonlinearity approximation, cascaded system, flexible joint

## Abstract

Control design for the nonlinear cascaded system is challenging due to its complicated system dynamics and system uncertainty, both of which can be considered some kind of system nonlinearity. In this paper, we propose a novel nonlinearity approximation scheme with a simplified structure, where the system nonlinearity is approximated by a steady component and an alternating component using only local tracking errors. The nonlinearity of each subsystem is estimated independently. On this basis, a model-free adaptive control for a class of nonlinear cascaded systems is proposed. A squared-error correction procedure is introduced to regulate the weight coefficients of the approximation components, which makes the whole adaptive system stable even with the unmodeled uncertainties. The effectiveness of the proposed controller is validated on a flexible joint system through numerical simulations and experiments. Simulation and experimental results show that the proposed controller can achieve better control performance than the radial basis function network control. Due to its simplicity and robustness, this method is suitable for engineering applications.

## 1. Introduction

The control problem of nonlinear cascaded systems commonly exists in engineering. Mechanical joints in robot manipulators are driven by motor currents [1,2,3]. The path tracking control of mobile robots is realized by adjusting wheel velocities [4,5,6,7]. Gyroscopic precession can be integrated into one-wheeled robots for steering control [8]. Flight dynamics in unmanned aerial vehicles (UAVs) can be stabilized through attitude adjustment [9,10,11,12]. Although these systems vary in physical assumptions, all of them can be modeled as nonlinear systems with a cascaded structure. The control design for such systems is challenging due to complicated system nonlinearity and uncertainty.

Disturbance rejection is a common approach to addressing the effects of unknown system nonlinearity and uncertainty. References [13,14] apply H-infinity optimal control for the linear system to suppress the effects of unknown disturbances. However, for systems with strong uncertainties, linear H-infinity control may lead to conservative performance. Hence, some researchers develop H-infinity controllers based on nonlinear system models [15,16,17]. Compared to the linear version, nonlinear H-infinity control allows for greater system nonlinearity under fine-tuning conditions and can delay control degradation and instability risks [17]. However, solving for nonlinear H-infinity controllers is usually complex and time-consuming [15,17,18]. In addition, invariant ellipsoid techniques are also introduced to optimize the robustness of control systems to unknown disturbances [19]. The invariant ellipsoid method simplifies the optimal controller to finding the smallest invariant ellipsoid of the closed-loop dynamic system [20]. A typical way is to apply the invariant ellipsoid method to suppress persistent disturbances through state-feedback control via LMI techniques [21,22,23]. It needs to quantitatively evaluate the effects of disturbances on the system output; thus, accurate system information is required. Other methods, such as the generalized fractional equation [24], are also introduced to model complex, uncertain systems.

Obtaining optimal control solutions for nonlinear systems with complex uncertainties is often challenging. Hence, researchers have proposed to combine the aforementioned disturbance rejection methods with nonlinearity estimation approaches that are free from system models, such as artificial neural networks (ANN) [25,26], fuzzy networks [27], and disturbance observers [18,28,29].

Artificial intelligence networks, such as fuzzy systems and neural networks, are commonly used for nonlinearity approximation [30,31,32,33,34,35,36,37]. In [31], a fuzzy approximation-based adaptive backstepping controller was developed to assist in the movement of an upper-limb exoskeleton robot. References [33,34,35] present observer-based fuzzy neural-network output feedback control algorithms for underactuated nonlinear systems. These studies combine the adaptive backstepping technique with artificially intelligent networks to achieve a high-performance approximation-based controller. Reference [38] proposes a reinforcement learning-based method to ensure asymptotic tracking control of continuous-time systems. However, the application of these approaches is hindered by complex control objects with a high degree of freedom (DOF), structural uncertainty, and system nonlinearity [36]. For artificial intelligence networks with complex topological structures, the learning process degrades the transient performance of the system and requires high calculation efficiency. For real-time control systems, their high computational cost is an inevitable challenge. References [32,36,37] stated that these factors impede the development of intelligence networks-based adaptive control, especially in real-time control applications.

High-gain disturbance observer (HGDOB) and sliding mode control (SMC) are also effective methods to deal with systems with parametric uncertainties and unmodeled nonlinearities. In [39], a HGDOB is designed to estimate the system disturbance caused by friction, load force, and the parameter disturbance for electro-hydraulic systems. However, the high gain observer is sensitive to measurement noise and delayed outputs [40]. To solve this problem, Reference [41] designed time-varying gains relying on the generalization of the Halanay-type inequalities. Reference [42] tried to lower the observer gain by introducing artificial delays and Taylor’s series. Similarly, the SMC is limited by chattering and peak phenomena in control signals [43]. In [44], a radial basis function neural network (RBFNN)-based soft computing strategy is applied to avoid the high switching gain that leads to chattering amplification. In [45], an adaptive sliding mode control method (ASMC) for robot manipulators is introduced. It utilizes the Taylor expansion to achieve a less conservative sign-function gain that enables chattering attenuation. The above approaches reduce chattering by applying extra-complicated policies. An interesting work is presented in [46] that presents a finite-time SMC (FT-SMC) and suppresses the peak phenomenon and chattering with an asymptotically convergent differentiator.

As can be seen from the previous discussion, in order to deal with unknown disturbances while avoiding problems caused by high control gains, controllers tend to become more and more complex and bloated. It is particularly unfriendly for engineering applications. Therefore, a simplified controller that is robust to unknown system nonlinearities and possesses mild control input is valuable for engineering applications.

Hence, this study aims to provide a simplified adaptive controller for a class of nonlinear cascaded systems. We first propose a so-called non-interference nonlinearity approximation (NINA) technique. It is based on the following system theory: For stable closed-loop systems, a bounded and continuous system nonlinearity can always be decomposed into steady and alternating components [47]. Furthermore, the output errors incorporated information relating to the system nonlinearity. Therefore, the unknown system nonlinearity can be modeled as a hierarchical form of a steady component and an alternating component. In addition, each nonlinearity can be approximated independently, using only local tracking errors. Thus, the proposed scheme is called non-interference nonlinearity approximation. Due to the simplified and decoupled approximation structure, the computational complexity of NINA is significantly reduced. Based on NINA, a model-free adaptive control is proposed. It is convenient for engineering applications because it avoids the fussy process of system modeling and parameter identification. In addition, it is also robust to external disturbance and parameter perturbation due to accurate nonlinearity approximation and compensation, which are verified by numerical simulations and experiments. Finally, its control inputs are milder than those of SMC and HGDOB-based control.

In summary, the contributions of this work are as follows:

(1) A novel NINA scheme that has a simplified hierarchical structure is proposed. Based on only local tracking errors, the NINA technique can approximate the unknown system nonlinearity regardless of its internal complexity. Saturation functions with adjustable shaping factors help balance fast convergence against measurement noise, thereby providing a mild control input.
(2) A model-free adaptive control based on the NINA technique is proposed. Its uniformly ultimate boundedness (UUB) is proven by the Lyapnuov theory. The effectiveness and robustness have been validated by simulations and experiments on a flexible-joint manipulator system.
(3) Compared with the intelligence network-based control, the proposed method possesses a simplified structure and requires less computational costs. Compared with the SMC, the proposed method can perform fast trajectory tracking with mild control inputs. Hence, it is convenient for engineering applications.

Reference [48] introduces an adaptive weighted saturation function to suppress system uncertainty in a stabilization problem. The approach was applied to flexible manipulator control by [49,50]. Different from previous work, this paper approximates the nonlinearity of the closed-loop system using trajectory tracking errors instead of relying on system states. Furthermore, a hierarchical approximation structure is introduced in this paper. The steady component aims to achieve fast tracking for the major part of the nonlinearity, while the alternating component is designed to supplementarily track its high-frequency fluctuations. In addition, this paper conducted an elaborate theoretical analysis that not only proves the effectiveness of the proposed approximation method but also provided the upper bound of the approximation error. The convergence of the weighted parameters was also analyzed. Hence, this work can be viewed as an extension of the approach in [48] to some degree.

The remainder of this paper is organized as follows: Section 2 formulates the dynamic model for a class of nonlinear cascaded systems. Section 3 presents a decoupled control framework. Section 4 describes the NINA technique. On this basis, Section 5 proposes NINA-based adaptive control. Numerical simulations and experiments on the flexible joint system are presented in Section 6 and Section 7, respectively. Conclusions are provided in Section 8.

## 2. Preliminaries

### Mathematical Description of the Generalized Dynamics

First, we consider a class of nonlinear cascaded systems with n-DOF whose dynamics are given by:

(1) $$\left[\begin{array}{cc} m_{\alpha }\left(\alpha ,\beta \right) & m_{\alpha \beta }\left(\alpha ,\beta \right)\\ m_{\beta \alpha }\left(\alpha ,\beta \right) & m_{\beta }\left(\alpha ,\beta \right) \end{array}\right]\left[\begin{array}{c} \ddot{\alpha }\\ \ddot{\beta } \end{array}\right]+\left[\begin{array}{c} n_{\alpha }\left(\alpha ,\dot{\alpha },\beta ,\dot{\beta }\right)\\ n_{\beta }\left(\alpha ,\dot{\alpha },\beta ,\dot{\beta }\right) \end{array}\right]=\left[\begin{array}{c} 0\\ \tau_{\beta } \end{array}\right]$$

where $\alpha ,\beta \in R^{n}$ represent the coordinates. $m_{\alpha },m_{\alpha \beta },m_{\beta \alpha },m_{\beta }\in R^{n\times n}$ form the system inertia matrix. $n_{\alpha },n_{\beta }\in R^{n}$, represent the system nonlinearity that captures centrifugal and Coriolis forces, viscous and frictions, gravitation, unmodeled system dynamics, and external disturbances. $\tau_{\beta }\in R^{n}$ represents the control inputs. The first and second rows in (1) represent the unactuated and actuated subsystems, respectively. For the convenience of distinguishing, they are denoted as the α-system and β-system.

For cascaded systems [1,2,3,4,5,6,7,8,9,10,11,12], the nonlinearity of the α-system $n_{\alpha }$ usually contains a dynamic coupling term that coordinates the behavior of the actuated and unactuated subsystems. Hence, $n_{\alpha }$ can be modeled as the combination of a known dynamic coupling term and a residual term, i.e.,

(2) $$n_{\alpha }\left(\alpha ,\dot{\alpha },\beta ,\dot{\beta }\right)=\delta_{\alpha }\left(\alpha ,\dot{\alpha },\beta ,\dot{\beta }\right)-u_{\alpha }\left(\alpha ,\dot{\alpha },\beta ,\dot{\beta }\right)$$

where $u_{\alpha }$ is the known dynamic coupling term and $\delta_{\alpha }$ is the unmolded system nonlinearity.

Substituting (2) into (1), the dynamic model can be represented as

(3) $$\left[\begin{array}{cc} m_{\alpha }\left(\alpha ,\beta \right) & m_{\alpha \beta }\left(\alpha ,\beta \right)\\ m_{\beta \alpha }\left(\alpha ,\beta \right) & m_{\beta }\left(\alpha ,\beta \right) \end{array}\right]\left[\begin{array}{c} \ddot{\alpha }\\ \ddot{\beta } \end{array}\right]+\left[\begin{array}{c} \delta_{\alpha }\left(\alpha ,\dot{\alpha },\beta ,\dot{\beta }\right)\\ n_{\beta }\left(\alpha ,\dot{\alpha },\beta ,\dot{\beta }\right) \end{array}\right]=\left[\begin{array}{c} u_{\alpha }\left(\alpha ,\dot{\alpha },\beta ,\dot{\beta }\right)\\ \tau_{\beta } \end{array}\right]$$

where the behavior of the α-system is indirectly regulated by the dynamic coupling term $u_{\alpha }$. Given the states of the α-system, the value of $u_{\alpha }$ depends on the states of the β-system. Therefore, the control objective is to perform trajectory tracking control of the α-system by regulating the behavior of the β system.

**Assumption** **1.** *Let* $x=\left(\alpha ,\dot{\alpha }\right)$*,* $y=\left(\beta ,\dot{\beta }\right)$*. For any given * $y_{1},y_{2}\in R^{n}$*, * $u_{\alpha }$ *satisfies the following Lipschitz condition:*

(4) $$\left\|u_{\alpha }\left(x,y_{2}\right)-u_{\alpha }\left(x,y_{1}\right)\right\|\leq \gamma \left\|y_{2}-y_{1}\right\|,$$

*where* γ>0 *is a finite constant.*

**Assumption** **2.** *Let* $S_{1}$*,*$\;S_{2}$*, and * $S_{3}$ *be the ranges of* x*,* y*, and * $u_{\alpha }$*, respectively. We have *$u_{\alpha }:(S_{1}\times S_{2})\to S_{3}$. *Given* $x\in S_{1}$*, for any desired* $u_{\alpha }\in S_{3}$*, there exists* $y_{r}\in S_{2}$ *satisfying the following inverse mapping:*

(5) $$u_{\alpha }^{-1}:(u_{\alpha r}\times x)\to y_{r},$$

*where* $u_{\alpha r}$ *is the desired value of* $u_{\alpha }$*, and* $y_{r}$ *is the desired value of* y*. This assumption is summarized from real systems* [1,2,3,4,5,6,7,8,9,10,11,12].

**Remark** **1.** *Assumptions 1 and 2 guarantee the maneuverability of the* α*-system. If we take* $u_{\alpha }\in S_{3}$ *as the virtual control and using (4) and (5), the error between* $u_{\alpha r}$ *and* $u_{\alpha }$ *is bounded by*

(6) $$\left\|u_{\alpha }\left(x,y_{r}\right)-u_{\alpha }\left(x,y\right)\right\|\leq \gamma \left\|y-y_{r}\right\|.$$

*We have* $u_{\alpha }\left(x,y\right)\to u_{\alpha }\left(x,\;y_{r}\right)$ *as* $y\to y_{r}$*. It indicates that the* α*-system can be indirectly regulated by the* β*-system via the dynamic coupling term* $u_{\alpha }$.

**Remark** **2.** *Equations (1) and (2) with assumptions 1 and 2 represent a class of nonlinear cascaded systems where the unactuated subsystems are indirectly regulated by the behaviors of the actuated subsystems through dynamic coupling. Some examples are provided as follows: For the flexible joint manipulator, the flexibility torque connects the dynamic behavior of the load and motor sides [3]. The gyro moment is used to maintain the lateral balance of the gyroscopic pendulum robot [7]. Dynamic coupling between attitude regulation torque and thrust force is widely utilized for the path tracking control of UAVs [9,10,11,12]. In the above examples, flexibility torque, the gyro moment, and aerodynamics can be viewed as the known dynamic coupling terms that can be used for controller design.*

## 3. Decoupled Control Framework

**Figure 1:**
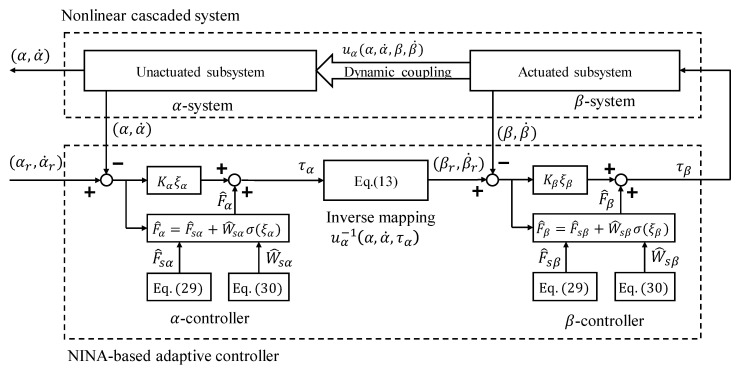
Decoupled cascaded control framework.

Considering system (1), there are two types of dynamic coupling: first, the dynamic coupling between the actuated and unactuated subsystems; and second, the dynamic coupling between different degrees of freedom (DOFs). To address the problem mentioned above, a decoupled control framework is proposed in this paper, as shown in Figure 1. To deal with the dynamic coupling between the actuated and unactuated subsystems, we introduce a cascaded control framework where an α-controller is placed in the outer layer to stabilize the unactuated subsystem and a β-controller is positioned in the inner layer to regulate the actuated subsystem. The two sub-controllers are linked through the inverse mapping of the dynamic coupling term $u_{\alpha }$. In addition, the dynamic coupling between different DOFs is considered to be an unknown disturbance and is compensated by the proposed NINA technique presented in the next section.

The control framework is derived below. Let $\alpha_{r}(t)$ and $\beta_{r}(t)$ be the reference trajectory of the α- and β-systems, which are assumed to be bounded and to have finite first- and second-order time derivatives. Let $e_{\alpha }=\alpha_{r}-\alpha$ and $e_{\beta }=\beta_{r}-\beta$ be the position tracking errors. Then, the following synthetic tracking errors are introduced:

(7) $$\begin{array}{c} \xi_{\alpha }=\Lambda_{\alpha }e_{\alpha }+{\dot{e}}_{\alpha }\\ \xi_{\beta }=\Lambda_{\beta }e_{\beta }+{\dot{e}}_{\beta } \end{array}.$$

where $\Lambda_{\alpha },\Lambda_{\beta }>0$ are diagonal positive gain matrices. Substituting (7) into model (3) and applying $\tau_{\alpha }=u_{\alpha r}$ as the virtual control, the error dynamics can be expressed as

(8) $$\left[\begin{array}{cc} m_{\alpha }\left(\alpha ,\beta \right) & m_{\alpha \beta }\left(\alpha ,\beta \right)\\ m_{\beta \alpha }\left(\alpha ,\beta \right) & m_{\beta }\left(\alpha ,\beta \right) \end{array}\right]\left[\begin{array}{c} \dot{\xi_{\alpha }}\\ {\dot{\xi }}_{\beta } \end{array}\right]=\left[\begin{array}{cc} m_{\alpha }\left(\alpha ,\beta \right) & m_{\alpha \beta }\left(\alpha ,\beta \right)\\ m_{\beta \alpha }\left(\alpha ,\beta \right) & m_{\beta }\left(\alpha ,\beta \right) \end{array}\right]\left[\begin{array}{c} \Lambda_{\alpha }{\dot{e}}_{\alpha }+{\ddot{\alpha }}_{r}\\ \Lambda_{\beta }{\dot{e}}_{\beta }+{\ddot{\beta }}_{r} \end{array}\right]+\left[\begin{array}{c} v_{\alpha }-\tau_{\alpha }\\ n_{\beta }-\tau_{\beta } \end{array}\right],\;$$

where $v_{\alpha }=\delta_{\alpha }+{\tilde{u}}_{\alpha }$ represents a lumped nonlinearity. ${\tilde{u}}_{\alpha }=u_{\alpha r}-u_{\alpha }$ is the distortion between the desired control input and its actual value. Such a distortion is mainly caused by state tracking errors, parameter perturbations, and the model uncertainty of $u_{\alpha }$.

Let us analyze (8) by choosing the following Lyapunov function:

(9) $$V_{1}=\frac{1}{2}\xi^{T}M\xi ,$$

with

$$\xi =\left[\begin{array}{c} \xi_{\alpha }\\ \xi_{\beta } \end{array}\right],M=\left[\begin{array}{cc} m_{\alpha }\left(\alpha ,\beta \right) & m_{\alpha \beta }\left(\alpha ,\beta \right)\\ m_{\beta \alpha }\left(\alpha ,\beta \right) & m_{\beta }\left(\alpha ,\beta \right) \end{array}\right].$$

Considering the time derivative of (9) and substituting (8), we obtain

(10) $${\dot{V}}_{1}=\xi^{T}\left(F-\tau \right),$$

with

$$\tau =\left[\begin{array}{c} \tau_{\alpha }\\ \tau_{\beta } \end{array}\right],$$

(11) $$F=\left[\begin{array}{c} F_{\alpha }\\ F_{\beta } \end{array}\right]=\frac{1}{2}\left[\begin{array}{cc} {\dot{m}}_{\alpha }\left(\alpha ,\beta \right) & {\dot{m}}_{\alpha \beta }\left(\alpha ,\beta \right)\\ {\dot{m}}_{\beta \alpha }\left(\alpha ,\beta \right) & {\dot{m}}_{\beta }\left(\alpha ,\beta \right) \end{array}\right]\left[\begin{array}{c} \xi_{\alpha }\\ \xi_{\beta } \end{array}\right]+\left[\begin{array}{cc} m_{\alpha }\left(\alpha ,\beta \right) & m_{\alpha \beta }\left(\alpha ,\beta \right)\\ m_{\beta \alpha }\left(\alpha ,\beta \right) & m_{\beta }\left(\alpha ,\beta \right) \end{array}\right]\left[\begin{array}{c} \Lambda_{\alpha }{\dot{e}}_{\alpha }+{\ddot{\alpha }}_{r}\\ \Lambda_{\beta }{\dot{e}}_{\beta }+{\ddot{\beta }}_{r} \end{array}\right]+\left[\begin{array}{c} v_{\alpha }\\ n_{\beta } \end{array}\right]$$

where F is an integrated system nonlinearity. Considering F as an unknown disturbance and compensating via nonlinearity estimation, a simplified control law can then be designed as

(12) $$\tau =K\xi +\hat{F},\;$$

where K is a positive, definite diagonal gain matrix. $\hat{F}$ is the estimation of F applied for nonlinearity compensation.

Substituting (12) into (10), ${\dot{V}}_{1}$ becomes

(13) $${\dot{V}}_{1}=-\xi^{T}K\xi +\xi^{T}(F-\hat{F}).$$

Ideally, if $\hat{F}=F$, ξ is asymptotically convergent to zero. If $\tilde{F}=F-\hat{F}$ is bounded, ξ will be ultimately bounded. It can be seen that the stability of the closed-loop system is determined by the nonlinearity approximation process. In the next section, a simplified NINA technique is proposed for the nonlinearity approximation.

**Remark** **3.** *As shown in* Figure 1*, the reference of the* α*-system* $\left(\alpha_{r},{\dot{\alpha }}_{r}\right)$ *is given by users, while the reference of the* β*-system* $\left(\beta_{r},{\dot{\beta }}_{r}\right)$ *is generated to guide the tracking of the virtual control* $u_{\alpha }\to u_{\alpha r}=\tau_{\alpha }$.

*Given the states of the* α*-system (*$\alpha ,\dot{\alpha })$ *and the desired value of virtual control,* $u_{\alpha r}$*, we have*

(14) $$u_{\alpha }\left(\alpha ,\dot{\alpha },\beta ,\dot{\beta }\right)\to u_{\alpha r}\left(\alpha ,\dot{\alpha },\beta_{r},{\dot{\beta }}_{r}\right)\;as\;\left(\beta ,\dot{\beta }\right)\to \left(\beta_{r},{\dot{\beta }}_{r}\right).\;$$

*Hence,* $\left(\beta_{r},{\dot{\beta }}_{r}\right)$ *can be obtained by solving the inverse mapping of* $u_{\alpha }(\alpha ,\dot{\alpha },\beta_{r},{\dot{\beta }}_{r})$ *with respect to* $\left(\beta_{r},{\dot{\beta }}_{r}\right)$*, i.e.,*

(15) $$(\beta_{r},{\dot{\beta }}_{r})=u_{\alpha }^{-1}\left(\alpha ,\dot{\alpha },u_{\alpha r}\right).$$

*An example of such inverse mapping about the elastic torque of the flexible joint manipulator is given in Equation (51).*

## 4. Principles of NINA

In this section, a simplified nonlinearity approximation scheme is presented. The nonlinearity of each subsystem can be estimated independently by simply utilizing the local tracking error.

**Declaration** **1.** *Considering the nonlinearity approximation by each subsystem, we adopt the following symbolic notation: for a vector V or a diagonal matrix V, the j-th element is marked by $V_{j}$, where j = 1, 2, …, 2n.*

Let $F_{j}$ represents the system nonlinearity and is assumed to be a bounded continuous time function. In real-world applications, most of the plants are controlled by digital controllers. Therefore, $F_{j}$ can be viewed as a piecewise time-varying function within successive control cycles. Mathematically, such a piecewise time-varying function can always be expressed as the synthetic form of steady and alternating components [47]. Hence, the system nonlinearity can be modeled in the time-domain as

(16) $$F_{j}\left(t\right)\equiv F_{sj}+\Delta_{j}\left(t\right),\left(t_{I}\leq t\leq t_{I}+\mu T_{c}\right)$$

where μ>1 is a positive integer. $t_{I}$ is the initial moment, and $T_{c}$ is the control cycle. $F_{sj}$ and $\Delta_{j}$ denote a bounded steady component and a bounded alternating component, respectively.

In addition, for closed-loop control systems, the tracking error reflects the combined effect of system nonlinearities. Therefore, we introduce the following structure to approximate system nonlinearities with the synthetic tracking error $\xi_{j}$:

(17) $$F_{Aj}\left(\xi_{j}\right)=F_{sj}+W_{sj}\sigma \left(\xi_{j}\right),\;\left(\left|\Delta_{j}\right|<W_{sj}<\infty \right),\;$$

where $F_{Aj}$ is the approximation of $F_{j}$. $F_{sj}$ is the steady component in (16), and $W_{sj}\sigma \left(\xi_{j}\right)$ is introduced to approximate the alternating component $\Delta_{j}$, where $W_{sj}$ is a dynamically adjusted weight coefficient and $\sigma \left(\xi_{j}\right)$ is a saturation function expressed as

(18) $$\sigma \left(\xi_{j}\right)=\xi_{j}/\left(\vartheta_{sj}+\left|\xi_{j}\right|\right),\;\left(0<\vartheta_{sj}<\infty \right),\;$$

where $\vartheta_{sj}$ is a shaping factor. When $\vartheta_{sj}\to 0$, $\sigma \left(\xi_{j}\right)$ acts as a signed switching function that is highly sensitive to the variation of $\xi_{j}$ around zero. By contrast, when $\vartheta_{sj}\to \infty$, $\sigma \left(\xi_{j}\right)$ tends to zero and becomes insensitive to the changes of $\xi_{j}$. Compared with the linear or polynomial approximation, the introduced saturation function enables a wide range of sensitivity adjustment w.r.t. $\xi_{j}$ via only one parameter. It is more convenient and adaptable.

From (16) and (17), the approximation error between $F_{j}\left(t\right)$ and $F_{Aj}$ can be calculated as

(19) $$E\left(\Delta_{j},\xi_{j}\right)=F_{j}\left(t\right)-F_{Aj}\left(\xi_{j}\right)=\Delta_{j}\left(t\right)-W_{sj}\sigma \left(\xi_{j}\right).\;$$

**Theorem** **1.** *For a bounded continuous nonlinearity* $F_{j}\left(t\right)$*, there exist optimized parameters* $F_{sj}$*,* $W_{sj}$*, and* $\vartheta_{sj}$ *for the approximation structure (17), (18) that satisfy the identity* $E\left(\Delta_{j},\xi_{j}\right)=0$ *and the synthetic tracking error is ultimately bounded by*

(20) $$\left|\xi_{j}\right|\leq \vartheta_{sj}\left(\left|\Delta_{j}/W_{sj}\right|\right)/\left[1+\left|\Delta_{j}/W_{sj}\right|\right],$$

*where* $\left|\xi_{j}\right|\to 0$*, as* $\vartheta_{sj}\to 0$ *and* $W_{sj}>\left|\Delta_{j}\right|$*. It illustrates that the proposed structure can effectively approximate the system nonlinearity while maintaining a small synthetic tracking error.*

**Proof** **.** Let ${\hat{F}}_{j}\left(t\right)=F_{A}\left(\xi_{j}\right)$ and substitute (19) into (13). The first derivative of the Lyapunov function in (13) becomes

(21) $${\dot{V}}_{1}=-\sum_{j}K_{j}\xi_{j}^{2}+\sum_{j}E\left(\Delta_{j},\xi_{j}\right)\xi_{j},$$

where $-K_{j}\xi_{j}^{2}\leq 0$. The property of $E\left(\Delta_{j},\xi_{j}\right)\xi_{j}$ is discussed below.

**Figure 2:**
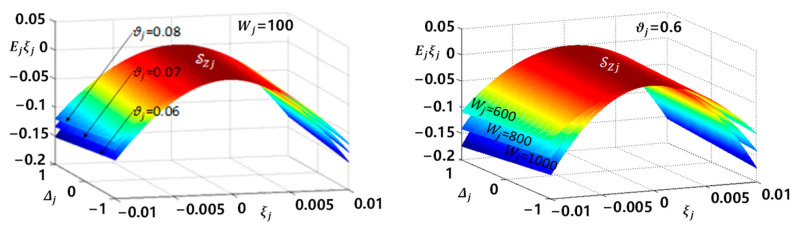
Changes in the profile of $E_{j}\left(\Delta_{j},\xi_{j}\right)\xi_{j}$ caused by parameter drifts.

*Step 1:* The second derivatives of $E\left(\Delta_{j},\xi_{j}\right)\xi_{j}$ with respect to $\xi_{j}$ is presented as

(22) $$\frac{\partial^{2}E\left(\Delta_{j},\xi_{j}\right)\xi_{j}}{\partial^{2}\xi_{j}}=2W_{sj}\left(\sigma \left(\xi_{j}\right)-1\right)\sigma^{\prime }\left(\xi_{j}\right),\;$$

where $\sigma^{\prime }\left(\xi_{j}\right)$ is the first derivatives of $\sigma \left(\xi_{j}\right)$ with respect to $\xi_{j}$. It can be verified segmentally that $\frac{\partial^{2}E\left(\Delta_{j},\xi_{j}\right)\xi_{j}}{\partial^{2}\xi_{j}}<0$ for any $\xi_{j}\in R$ and $W_{sj}>0$. Hence, $E\left(\Delta_{j},\xi_{j}\right)\xi_{j}$ is an open downward convex function with respect to $\xi_{j}$. This can be further verified by the profile diagram shown in Figure 2.

*Step 2:* Given that $E\left(\Delta_{j},\xi_{j}\right)\xi_{j}$ is an open downward convex function with respect to $\xi_{j}$, its sign will vary around the nonzero solution ${\xi_{j}=\xi }_{z}\left(\Delta_{j}\right)$ of equation $E\left(\Delta_{j},\xi_{j}\right)\xi_{j}=0$. With (19), the value of $\xi_{z}\left(\Delta_{j}\right)$ can be calculated as

(23) $$\xi_{z}\left(\Delta_{j}\right)=\left\{\begin{array}{c} \xi_{z}^{+}=\vartheta_{sj}\left(\Delta_{j}/W_{sj}\right)/\left[1-\left(\Delta_{j}/W_{sj}\right)\right],\left(\Delta_{j}\geq 0\right)\\ \xi_{z}^{-}=\vartheta_{sj}\left(\Delta_{j}/W_{sj}\right)/\left[1+\left(\Delta_{j}/W_{sj}\right)\right],\left(\Delta_{j}\leq 0\right) \end{array}\right.,\;$$

*Step 3:* The following two compact sets are defined accordingly:

$$S_{zj}^{+}≝\left\{\left(\xi_{j},\Delta_{j}\right)\in R^{2}\left|0<\xi_{j}<\xi_{z}^{+},\Delta_{j}>0\right.\right\},$$

$$S_{zj}^{-}≝\left\{\left(\xi_{j},\Delta_{j}\right)\in R^{2}\left|\xi_{z}^{-}<\xi_{j}<0,\Delta_{j}<0\right.\right\}.$$

*Step 4:* Substituting $E\left(\Delta_{j},\xi_{j}\right)\xi_{j}$ around the domain $S_{zj}^{+}\cup S_{zj}^{-}$, we can verify that, for $\left(\xi_{j},\Delta_{j}\right)\notin S_{zj}^{+}\cup S_{zj}^{-}$, there is

(24) $$E\left(\Delta_{j},\xi_{j}\right)\xi_{j}<0,\;$$

and for $\left(\xi_{j},\Delta_{j}\right)\in S_{zj}^{+}\cup S_{zj}^{-}$, there is

(25) $$E\left(\Delta_{j},\xi_{j}\right)\xi_{j}>0.\;$$

Using (21) and considering the extreme case when $K_{j}\to 0$, if $\left(\xi_{j},\Delta_{j}\right)\in S_{zj}^{+}\cup S_{zj}^{-}$, ${\dot{V}}_{1}$ tends to be positive and $\xi_{j}$ diverges from $S_{zj}^{+}\cup S_{zj}^{-}$. By contrast, if $\left(\xi_{j},\Delta_{j}\right)\notin S_{zj}^{+}\cup S_{zj}^{-}$, ${\dot{V}}_{1}<0$ and $\xi_{j}$ converges back to $S_{zj}^{+}\cup S_{zj}^{-}$. This variation proves that $\xi_{j}$ is ultimately restricted within $S_{zj}^{+}\cup S_{zj}^{-}$, which provides

(26) $$\left|\xi_{j}\right|\leq \vartheta_{sj}\left|\Delta_{j}/W_{sj}\right|/\left[1+\left|\Delta_{j}/W_{sj}\right|\right].$$

According to (23) and (26), if the candidates are chosen as $\vartheta_{sj}\ll 1$ and $W_{sj}\gg \left|\Delta_{j}\right|$, there exists $\xi_{z}\left(\Delta_{j}\right)$ that tends to zero and satisfies the identity: $E\left(\Delta_{j},\xi_{z}\left(\Delta_{j}\right)\right)\equiv 0.$ It illustrates that $F_{Aj}\left(\xi_{j}\right)$ in (17) can effectively approximate the system nonlinearity $F_{j}\left(t\right)$ around the domain $S_{zj}^{+}\cup S_{zj}^{-}$, while maintaining a small synthetic tracking error. □

## 5. Adaptive Control Based on NINA

In this section, an adaptive control utilizing NINA is fulfilled, and the stability analysis is carried out. 

### 5.1. Adaptive Law

Given that $F_{j}$ and $W_{sj}$ are optimal candidates for the approximation structure in (17), the integrated error $\iint E^{2}\left(\Delta_{j},\xi_{j}\right)d\Delta_{j}d\xi_{j}$ is minimized. The estimation of $F_{j}\left(t\right)$ is defined as

(27) $${\hat{F}}_{j}\left(t\right)={\hat{F}}_{Aj}\left(\xi_{j}\right)={\hat{F}}_{sj}+{\hat{W}}_{sj}\sigma \left(\xi_{j}\right).\;$$

where ${\hat{F}}_{sj}$ and ${\hat{W}}_{sj}$ are the estimations of $F_{j}$ and $W_{sj}$, respectively. Subtracting (27) from (16), the error between $F_{j}\left(t\right)$ and ${\hat{F}}_{j}\left(t\right)$ is represented as

(28) $${\tilde{F}}_{j}\left(t\right)={\tilde{F}}_{sj}+{\tilde{W}}_{sj}\sigma \left(\xi_{j}\right)+E\left(\Delta_{j},\xi_{j}\right),$$

with ${\tilde{F}}_{sj}=F_{sj}-{\hat{F}}_{sj}$ and ${\tilde{W}}_{sj}=W_{sj}-{\hat{W}}_{sj}.$ The adaptive law of ${\hat{F}}_{sj}$ and ${\hat{W}}_{sj}$ is given by the following squared-error correction procedures:

(29) $${\dot{\hat{W}}}_{sj}=-A_{j}\xi_{j}^{2}{\hat{W}}_{sj}+B_{j}\sigma \left(\xi_{j}\right)\xi_{j}\;$$

(30) $${\dot{\hat{F}}}_{sj}=-A_{j}\xi_{j}^{2}{\hat{F}}_{sj}+B_{j}\xi_{j}\;$$

where $A_{j},B_{j}>0$ are the adaptive gains. The term $A_{j}\xi_{j}^{2}$ plays a role in preventing the divergences of ${\hat{W}}_{sj}$ and ${\hat{F}}_{sj}$.

Using (29) and (30), the transient performance of ${\tilde{F}}_{sj}$ and ${\tilde{W}}_{sj}$ is analyzed next. Applying ${\dot{\tilde{W}}}_{sj}=-{\dot{\hat{W}}}_{sj}$ and ${\dot{\tilde{F}}}_{sj}=-{\dot{\hat{F}}}_{sj}$, (29) and (30) can then be represented as

(31) $${\dot{\tilde{W}}}_{sj}=-A_{j}\xi_{j}^{2}{\tilde{W}}_{sj}+\rho \left(\xi_{j}\right),\;$$

(32) $${\dot{\tilde{F}}}_{sj}=-A_{j}\xi_{j}^{2}{\tilde{F}}_{sj}+\varrho \left(\xi_{j}\right),\;$$

where

$$\rho =\left(A_{j}\xi_{j}W_{sj}-B_{j}\sigma \left(\xi_{j}\right)\right)\xi_{j},\varrho =\left(A_{j}\xi_{j}F_{sj}-B_{j}\right)\xi_{j}.$$

The solutions of (31) and (32) are represented as

(33) $${\tilde{W}}_{sj}\left(t\right)=\phi \left(t,t_{I}\right){\tilde{W}}_{sj}\left(t_{I}\right)+\int_{t_{I}}^{t}\phi \left(t,\tau \right)\rho \left(\tau \right)d\tau ,\;$$

(34) $${\tilde{F}}_{sj}\left(t\right)=\phi \left(t,t_{I}\right){\tilde{F}}_{sj}\left(t_{I}\right)+\int_{t_{I}}^{t}\phi \left(t,\tau \right)\varrho \left(\tau \right)d\tau ,\;$$

where

(35) $$\phi \left(t,t_{I}\right)=exp\left(-A_{j}\int_{t_{I}}^{t}\xi_{j}^{2}\left(\tau \right)d\tau \right).$$

Supposing that the persistent excitation condition holds for $\xi_{j}$*,* $\phi \left(t,t_{I}\right)$ asymptotically converges to zero with the increase in t, proving that (31) and (32) are bounded-input-bounded-output stable. The expressions of ρ and ϱ show that $\xi_{j}$ is a unique source that changes ${\tilde{W}}_{sj}$ and ${\tilde{F}}_{sj}$ in the steady state. Therefore, ${\tilde{W}}_{sj}$ and ${\tilde{F}}_{sj}$ can be restricted within a small area along with the convergence of $\xi_{j}$. We can also conclude from (35) that ${\tilde{W}}_{sj}$ and ${\tilde{F}}_{sj}$ obtain fast convergence as long as $A_{j}$ is set to make the steady-state time of $\phi \left(t,t_{I}\right)$ much smaller than $\mu T_{c}$. 

### 5.2. Adaptive Controller and Stability Analysis

Combining the control structure in (12), and approximating the system nonlinearity using (27), (29), and (30), the NINA-based adaptive control law is presented as

(36) $$\left\{\begin{array}{c} \tau_{j}=K_{j}\xi_{j}+{\hat{F}}_{sj}+{\hat{W}}_{sj}\sigma \left(\xi_{j}\right)\\ {\dot{\hat{W}}}_{sj}=-A_{j}\xi_{j}^{2}{\hat{W}}_{sj}+B_{j}\sigma \left(\xi_{j}\right)\xi_{j}\\ {\dot{\hat{F}}}_{sj}=-A_{j}\xi_{j}^{2}{\hat{F}}_{sj}+B_{j}\xi_{j} \end{array}\right..\;$$

**Theorem** **2.** *Consider the nonlinear system represented by (3) and use the control law in (36). If control gain*$K_{j}$ *is selected in accordance with the following inequality*

(37) $$K_{j}>\frac{1}{4}\left[A_{j}\left(W_{sj}^{2}+F_{sj}^{2}\right)/B_{j}\right],$$

*the synthetic tracking* $\xi_{j}$*is uniformly and ultimately bounded by*

$${\left|\xi_{j}\right|}^{2}\leq W_{sj}\vartheta_{sj}/G_{j}$$

*with*

$$G_{j}=K_{j}-\frac{1}{4}\left[A_{j}\left(W_{sj}^{2}+F_{sj}^{2}\right)/B_{j}\right]$$

*Moreover, the synthetic tracking error* $\xi_{j}$*can be restricted to a small area around zero as*  $0<{(\vartheta }_{sj},A_{j})\ll 1$ *and* $(K_{j}$*,* $B_{j})\gg 1$*.*

**Proof.**  A Lyapunov function candidate is selected as

(38) $$V_{2}=V_{1}+\frac{1}{2B_{j}}\sum_{j}\left({\tilde{W}}_{sj}^{2}+{\tilde{F}}_{sj}^{2}\right),\;$$

Using (13) and differentiating (38) with respect to time, we have

(39) $${\dot{V}}_{2}=\sum_{j}\left[\xi_{j}{\tilde{F}}_{j}\left(t\right)+\left({\tilde{W}}_{sj}{\dot{\tilde{W}}}_{sj}+{\tilde{F}}_{sj}{\dot{\tilde{F}}}_{sj}\right)/B_{j}-K_{j}\xi_{j}^{2}\right],\;$$

Applying ${\dot{\tilde{W}}}_{sj}=-{\dot{\hat{W}}}_{sj}$ and ${\dot{\tilde{F}}}_{sj}=-{\dot{\hat{F}}}_{sj}$ with (29) and (30), ${\dot{V}}_{2}$ becomes

(40) $${\dot{V}}_{2}=\sum_{j}\left[\begin{array}{c} A_{j}\xi_{j}^{2}\left({\tilde{W}}_{sj}{\hat{W}}_{sj}+{\tilde{F}}_{sj}{\hat{F}}_{sj}\right)/B_{j}\\ +E_{j}\left(\Delta_{j},\xi_{j}\right)\xi_{j}-K_{j}\xi_{j}^{2} \end{array}\right].$$

where ${\tilde{W}}_{sj}{\hat{W}}_{sj}$ and ${\tilde{F}}_{sj}{\hat{F}}_{sj}$ are bounded by

(41) $${\tilde{W}}_{sj}{\hat{W}}_{sj}\equiv {\tilde{W}}_{sj}\left(W_{sj}-{\tilde{W}}_{sj}\right)\leq W_{sj}^{2}/4,\;$$

(42) $${\tilde{F}}_{sj}{\hat{F}}_{sj}\equiv {\tilde{F}}_{sj}\left(F_{sj}-{\tilde{F}}_{sj}\right)\leq F_{sj}^{2}/4.\;$$

Substituting (41) and (42) into (40), we can obtain the following inequality:

(43) $${\dot{V}}_{2}\leq -\sum_{j}G_{j}\xi_{j}^{2}+\sum_{j}E\left(\Delta_{j},\xi_{j}\right)\xi_{j},\;$$

where $G_{j}=K_{j}-\frac{1}{4}\left[A_{j}\left(W_{sj}^{2}+F_{sj}^{2}\right)/B_{j}\right]$.

According to the conclusion in (22) that $E\left(\Delta_{j},\xi_{j}\right)\xi_{j}$ is an open-downward convex function with respect to $\xi_{j}$, $E\left(\Delta_{j},\xi_{j}\right)\xi_{j}$ will reach its maximum value as

(44) $$\frac{\partial E\left(\Delta_{j},\xi_{j}\right)\xi_{j}}{\partial \xi_{j}}=0.$$

Substituting the solution of (44), the maximum value of $E\left(\Delta_{j},\xi_{j}\right)\xi_{j}$ can be calculated as

(45) $$E\left(\Delta_{j},\xi_{j}\right)\xi_{j}\leq W_{sj}\sigma^{2}\left(\xi_{j}\right)\vartheta_{sj}=W_{sj}\vartheta_{sj}\xi_{j}^{2}/{\left(\vartheta_{sj}+\left|\xi_{j}\right|\right)}^{2}.$$

Applying (45), (43) becomes

(46) $${\dot{V}}_{2}\leq -\sum_{j}G_{j}\xi_{j}^{2}+\sum_{j}\left[W_{sj}\vartheta_{sj}/{\left(\vartheta_{sj}+\left|\xi_{j}\right|\right)}^{2}\right]\xi_{j}^{2}.$$

If ${\left|\xi_{j}\right|}^{2}>\frac{W_{sj}\vartheta_{sj}}{G_{j}}$, we have ${\dot{V}}_{2}<0$. Hence, the synthetic tracking error $\xi_{j}$ is uniformly and ultimately bounded by

(47) $${\left|\xi_{j}\right|}^{2}\leq W_{sj}\vartheta_{sj}/G_{j}.$$

The synthetic tracking error $\xi_{j}$ will be restricted into a small area around zero as $0<{(\vartheta }_{sj},A_{j})\ll 1$ and $(K_{j}$,$B_{j})\gg 1$. This completes the proof. □

## 6. Numerical Simulation

In this section, the proposed method is verified by the trajectory tacking control of a two-link flexible-joint manipulator. The simulations of the manipulator under step change, different link lengths, and joint stiffness are performed to evaluate the robustness of the proposed method. The simulations are conducted utilizing the fourth-order Runge–Kutta method.

The finite-time sliding mode control (FT-SMC) in [46] and the RBFN-based control in [51] are also simulated for comparison. These two controllers are selected as representatives of sliding mode control and RBFN-based control methods. They exhibit relatively simple yet representative architectures and are also model-free methods, which makes them suitable as benchmarks for comparison.

### 6.1. Simulation Setup

**Figure 3:**
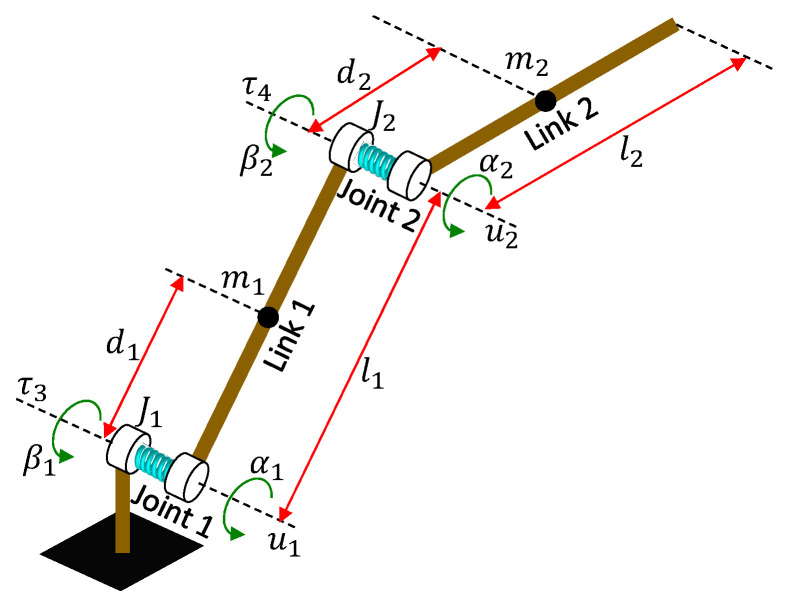
Architecture of the two-link robot manipulator with joint flexibility.

The configuration of the manipulator is depicted in Figure 3, and its parameters are listed in Table 1. Referring to [52], the system dynamics can be modeled as

(48) $$\left[\begin{array}{cc} m_{11}(\alpha_{1},\alpha_{2}) & m_{12}(\alpha_{1},\alpha_{2})\\ m_{21}(\alpha_{1},\alpha_{2}) & m_{22}(\alpha_{1},\alpha_{2}) \end{array}\right]\left[\begin{array}{c} {\ddot{\alpha }}_{1}\\ {\ddot{\alpha }}_{2} \end{array}\right]+\left[\begin{array}{c} h_{1}\\ h_{2} \end{array}\right]+\left[\begin{array}{c} G_{1}\\ G_{2} \end{array}\right]=\left[\begin{array}{c} u_{1}\\ u_{2} \end{array}\right]\;$$

(49) $$\left[\begin{array}{cc} J_{1}(\beta_{1}) & 0\\ 0 & J_{2}(\beta_{2}) \end{array}\right]\left[\begin{array}{c} {\ddot{\beta }}_{1}\\ {\ddot{\beta }}_{2} \end{array}\right]+\left[\begin{array}{c} f_{d1}\\ f_{d2} \end{array}\right]-\left[\begin{array}{c} u_{1}\\ u_{2} \end{array}\right]=\left[\begin{array}{c} \tau_{3}\\ \tau_{4} \end{array}\right]\;$$

with

(50) $$\left\{\begin{array}{c} u_{1}=k_{s1}\left(\alpha_{1}-\beta_{1}\right)+k_{d1}({\dot{\alpha }}_{1}-{\dot{\beta }}_{1})\\ u_{2}=k_{s2}\left(\alpha_{2}-\beta_{2}\right)+k_{d2}({\dot{\alpha }}_{2}-{\dot{\beta }}_{2}) \end{array}\right.$$

where $\alpha ={\left(\alpha_{1},\alpha_{2}\right)}^{T}$ and $\beta ={\left(\beta_{1},\beta_{2}\right)}^{T}$ are the position vectors of the load and motor sides, respectively; $m_{11},\;m_{12},m_{21},m_{22}$ are the elements of the load side inertial matrix, $J_{1}$ and $J_{2}$ are the elements of the motor side inertial matrix; $h_{1}$ and $h_{2}$ consist of Coriolis and centrifugal terms; $G_{1}$ and $G_{2}$ contain gravitational terms; $u_{1}$ and $u_{2}$ are the elastic torque terms;$\;f_{d1}$ and $f_{d2}$ are the damping terms of the motor side; and $\tau_{3}$ and $\tau_{4}$ are the motor torques.

The control law presented in Theorem 2 is applied. The reference trajectory of the load-side is given by the user command. The reference trajectory of the motor side is generated by solving the following differential equation:

(51) $$\left\{\begin{array}{c} {\dot{\beta }}_{r1}=-k_{s1}\beta_{r1}/k_{d1}+\left[\left(\tau_{\alpha 1}+k_{s1}\alpha_{1}\right)/k_{d1}+{\dot{\alpha }}_{1}\right]\\ {\dot{\beta }}_{r2}=-k_{s2}\beta_{r2}/k_{d2}+\left[\left(\tau_{\alpha 2}+k_{s2}\alpha_{2}\right)/k_{d2}+{\dot{\alpha }}_{2}\right] \end{array}\right.,$$

where $\left(\beta_{r1},\beta_{r2},{\dot{\beta }}_{r1},{\dot{\beta }}_{r2}\right)$ represent the command trajectory of the motor side, and $\tau_{\alpha 1}$ and $\tau_{\alpha 2}$ are the desired values of $u_{1}$ and $u_{2}$, respectively. This formula is an inverse mapping of $u_{1}(\alpha_{1},{\dot{\alpha }}_{1},\beta_{1r},{\dot{\beta }}_{1r})$ and $u_{2}(\alpha_{2},{\dot{\alpha }}_{2},\beta_{2r},{\dot{\beta }}_{2r})$ in (50). The parameters of the proposed adaptive controller used in the simulation are listed in Table 2.

**Remark** **4.** *For the flexible joint system mentioned above, the motor side is the actuated subsystem, and the link side is the unactuated subsystem. The elastic torque $\left(u_{1},u_{2}\right)$ helps coordinate the behavior of the motor and load sides. It can be verified that Assumptions 1 and 2 hold for $\left(u_{1},u_{2}\right)$ by examining the expression in (50).*

### 6.2. Trajectory Tracking Performance Validation

We compared the trajectory tracking performance of the three control methods: the proposed method, the RBFN-based method, and FT-SMC. The robot arm starts from the horizontal position and tracks sinusoidal trajectories as shown below:

$$\left\{\begin{array}{c} \alpha_{r}=\frac{\pi }{6}\left(1+\sin\left(2t\right)\right),load\;side\\ \beta_{r}=\frac{\pi }{6}\left(1+\cos\left(2t\right)\right),motor\;side \end{array}.\right.$$

**Figure 4:**
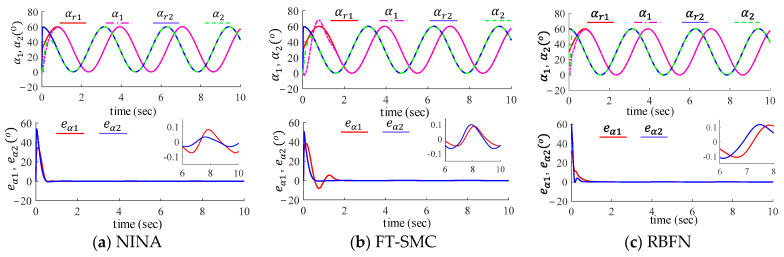
Link-side tracking performance of (**a**) the proposed method, (**b**) FT-SMC, and (**c**) RBFN.

To evaluate the tracking performance of the controllers, we introduce the following evaluation index, and the results are listed in Table 3 and Table 4:

(a) SSP (steady-state tracking error in position):
  $$SSP=\left[\min e_{j}(t)\;maxe_{j}\left(t\right)\right],\;for\;t>t_{M1},\;j=\alpha \;or\;\beta$$
  where $\min e_{j}(t)$ and $max\;e_{j}\left(t\right)$ represent the lower and upper bounds of the position tracking error, respectively. $t_{M1}$ represents the time since the tracking error varied periodically and steadily. In this simulation, it is set $t_{M1}=4\;s$.
(b) CTP (convergence time of trajectory tracking): It is defined as the time when the tracking error is free from initial oscillation, shown in Figure 4c, and first comes into the range of steady-state, i.e., SSP.
(c) SSE (steady-state estimation error in system nonlinearity):
  $$SSE=\left[\min{\tilde{F}}_{j}\left(t\right)\;\max{\tilde{F}}_{j}\left(t\right)\right],for\;t>t_{M2},\;j=\alpha \;or\;\beta$$
  where $\min{\tilde{F}}_{j}\left(t\right)$ and $\max{\tilde{F}}_{j}\left(t\right)$ represent the low and up bounds of the nonlinearity estimation error, respectively. $t_{M2}$ is defined similarly to $t_{M1}$ and is set as $t_{M2}=4\;s$.
(d) CTE (convergence time of nonlinearity estimation): It is similar to the definition of CTP.

**Figure 5:**
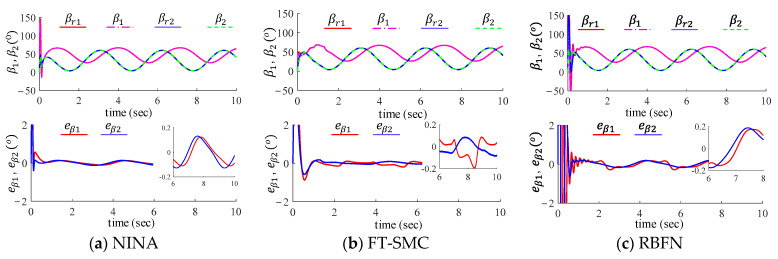
Motor-side tracking performance of (**a**) the proposed method, (**b**) FT-SMC, and (**c**) RBFN.

The tracking performance of the three controllers is illustrated in Figure 4 and Figure 5. The steady-state tracking accuracy and convergence time are listed in Table 3. All three controllers can effectively track sinusoidal trajectories. Among them, the proposed algorithm exhibits smooth and fast convergence during the transient phase, while the other two methods show more pronounced oscillations. This is due to excessive control gain. As shown in Table 3, the tracking errors of the proposed control algorithm on the load side for joints 1 and 2 are, respectively −0.07°~0.08° and −0.03°~0.03°; on the motor side, the tracking errors are −0.11°~0.12° and −0.13°~0.13°, respectively. The overall steady-state tracking accuracy of the proposed algorithm is superior to the other two control algorithms.

**Figure 6:**
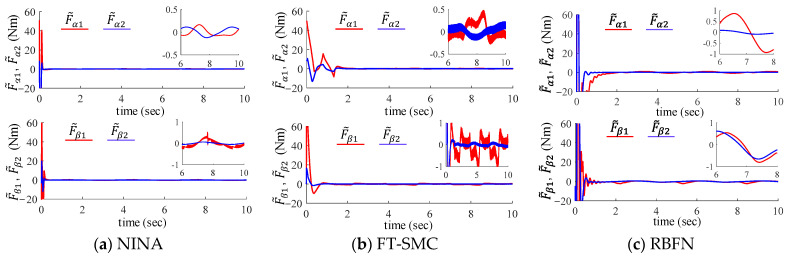
Approximation errors of the unknown system nonlinearity using (**a**) the proposed method, (**b**) the FT-SMC, and (**c**) the RBFN method.

It can be seen from Figure 6 and Table 4 that the proposed algorithm achieves faster convergence of the nonlinear approximation error than the other two methods. This verifies its ability to track unknown disturbances with high dynamics. In addition, the proposed method also illustrates the high estimation accuracy of system nonlinearity.

**Figure 7:**
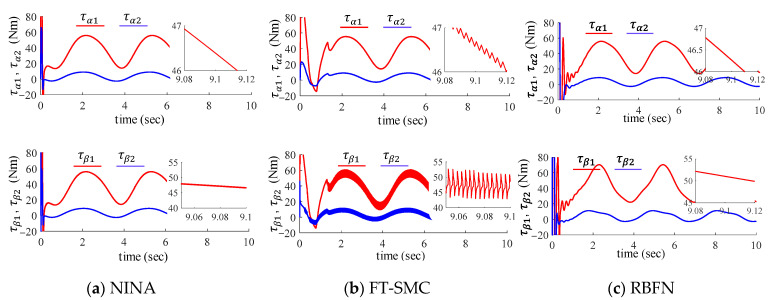
Control efforts of the proposed method (**top** row), the FT-SMC (**middle** column), and the RBFN method (**bottom** row).

Finally, the control signals of the three controllers are depicted in Figure 7. The control inputs of the proposed method and the RBFN-based method are milder, while the one of the FT-SMC shows significant chattering, especially on the motor side. It is a typical problem for sliding mode control. Compared with the traditional SMC method, the FT-SMC presented in [46] solved the peak phenomenon and suppressed the control chattering by asymptotical convergence, which is a considerable contribution. However, for nonlinear cascaded systems such as the flexible-joint manipulator, the control input of the outer loop (the load side) is usually mapped as the command of the inner loop (the motor side). This mapping process transmits the small chattering on the load side into the command layer of the motor side. The suppressed chattering is then amplified again by the motor-side control loop. As verified in Figure 7, the control input of the motor side contains obvious chattering, while the control input of the load side is milder.

In summary, the FT-SMC control shows good trajectory tracking accuracy and nonlinear estimation accuracy, but significant chattering occurs, which can lead to the failure of precision sensors and actuators in practical applications. Neural network-based control such as the RBFN-based method shows relatively lower convergence speed for nonlinearity approximation due to its comparably complex topology. In contrast, the proposed algorithm adopts a simple and effective estimation structure, which not only shows the ability for fast and accurate nonlinearity approximation but also maintains mild control input. This is also the major motivation for our research on this algorithm.

### 6.3. Robustness Validation

**Figure 8:**
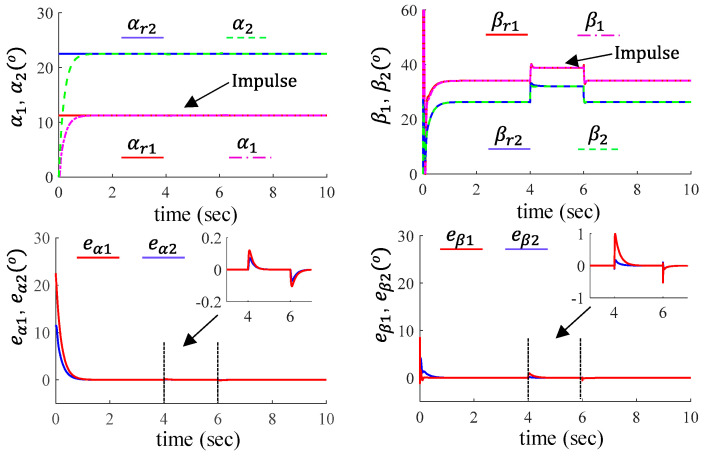
Tracking performance of the proposed method under step change and impulse disturbance. The first row shows the position of the load and motor sides, and the second row shows their tracking errors.

As shown in Figure 8, to further verify the stability and robustness of the proposed control, we examined the step response of the proposed method and its ability to recover from sudden disturbances. It can be observed that when encountering step changes, each joint can quickly track the new reference signal. The settling times for joints 1 and 2 are 0.618 s and 0.60 s, respectively. A 10 Nm impulse disturbance is introduced at 4 s and revoked at 6 s. It can be seen that the system can recover tracking of the original position within 2 s and has the ability to maintain a fixed point position with high precision (position tracking error $<1\times 10^{-5}$ degree).

**Figure 9:**
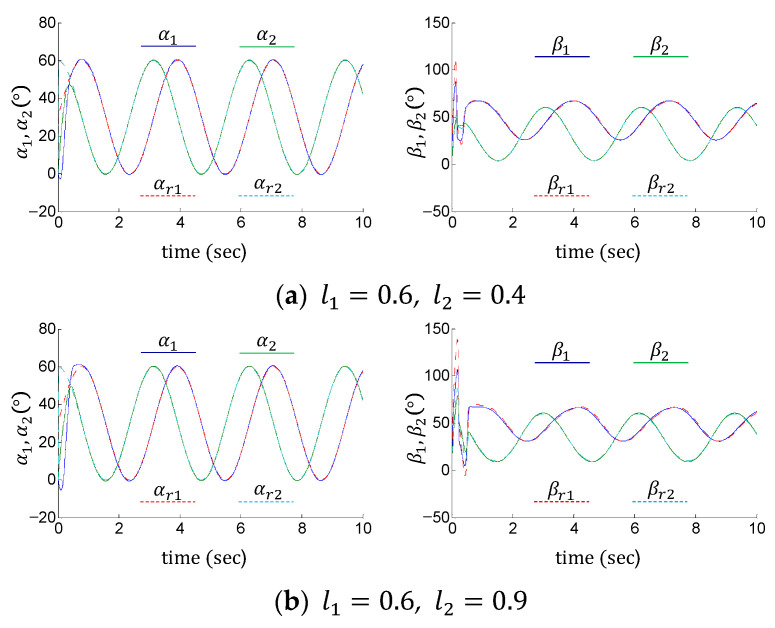
Results of tracking control under different link lengths using the proposed control method.

**Figure 10:**
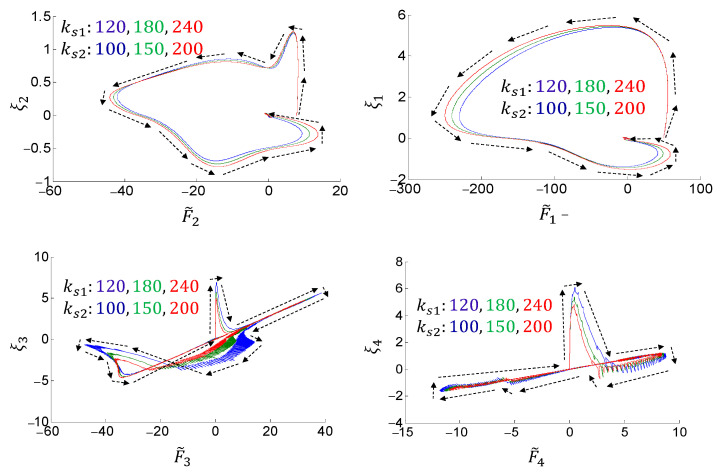
Dynamic behavior of the manipulator under different joint stiffness using the proposed control method.

Figure 9 compares the tracking performance of the proposed control method under different link lengths. Although the load environment has changed, the proposed adaptive control maintains high tracking performance. Figure 10 illustrates the dynamic behavior of the whole system under different joint stiffnesses. It is illustrated that all the synthetic tracking errors and nonlinearity estimation errors uniformly and asymptotically converge toward zero, regardless of the variation of the joint stiffness. These results verify the effectiveness and robustness of the proposed method.

## 7. Experiments

In this section, the proposed control method is further validated on a flexible-joint platform. The RBFN-based adaptive control method in [51] is introduced for comparison. Trajectory tracking experiments under different end loads are conducted.

### 7.1. Experiment Setup

**Figure 11:**
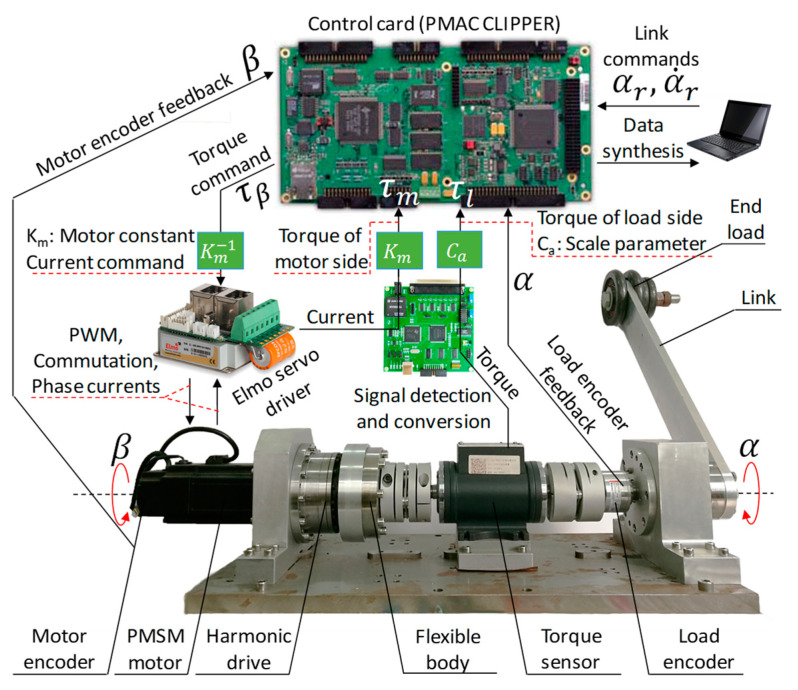
Architecture of the experimental system.

Figure 11 shows a typical flexible-joint platform. From the left to right sides, there are a servo motor, a harmonic drive (with a 50:1 gear ratio), a flexible body, a torque sensor, and an output link with an end load. The flexible body here is a series of elastic actuators. The angular positions of load side  α and motor side β are measured by optical encoders. The generated torque command $\tau_{\beta }$ is implemented through a servo driver. The torque sensor and signal detection-conversion card are employed to measure the output torque of load side $\tau_{l}$ and motor side $\tau_{m}$, respectively. The nominal parameters of the platform are obtained via parameter identification and measurement, which are listed in Table 5.

### 7.2. Experimental Results

**Figure 12:**
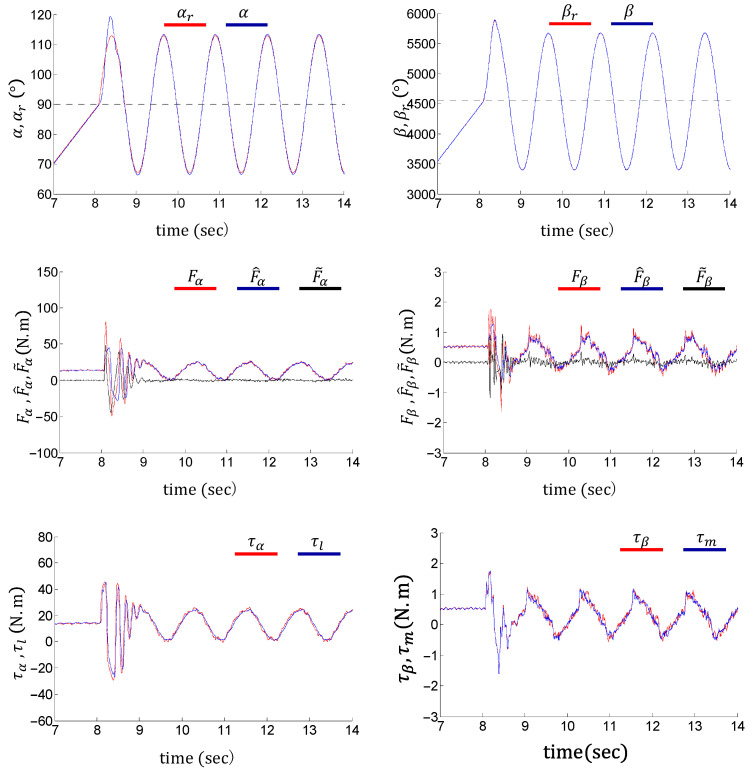
Performance of NINA-based adaptive control under a 2 kg end load.

**Figure 13:**
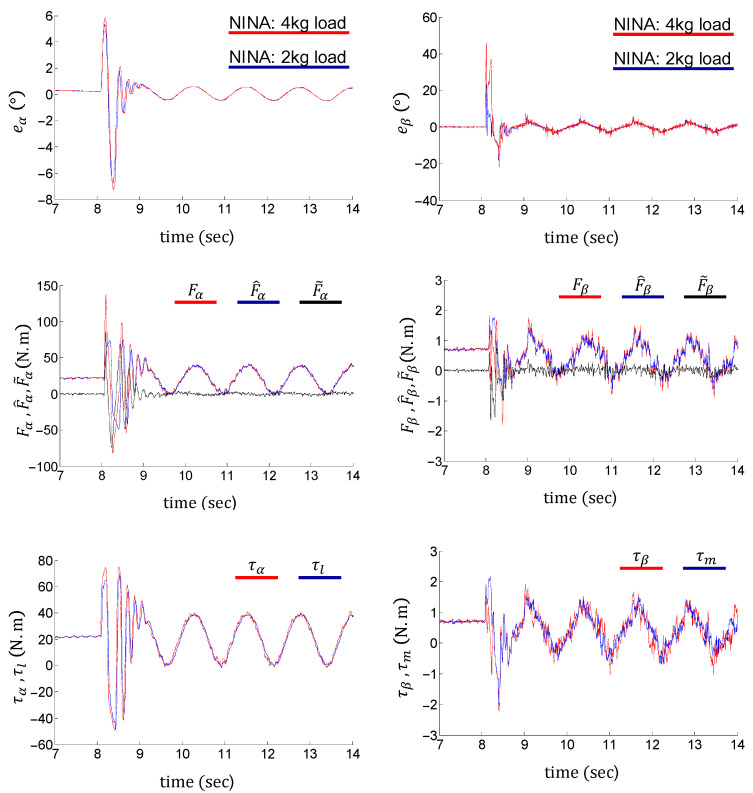
Performance of NINA-based adaptive control under a 4 kg end load.

Figure 12 and Figure 13 show the tracking performance, nonlinearity approximations, and control inputs of the flexible joint using the proposed control method under 2 kg and 4 kg load conditions, respectively. The link action is set as follows: The initial posture of the link is vertically downward. It first rotates at a constant speed of 18°/s toward the horizontal level, then swings around the horizontal position. The swing amplitude and frequency are 21.6° and 0.8 Hz, respectively. The black dotted lines in the left column of Figure 12 indicate the horizontal position. The experimental results verify the tracking performance of the control system under both ramp and harmonic trajectories. The entire process is divided into three phases, i.e., the ramping phase, the switching phase, and the waving phase. The system exhibits transient responses in the switching phase (8 s to 10 s), due to the discontinuity of the velocity command ${\dot{\alpha }}_{r}$.

As shown in the first rows of Figure 12 and Figure 13, the control system stabilizes within 1 s during the switching phase. Tracking errors are limited within 0.5° and 10° on the load and motor sides (with a 50:1 gear ratio) during the ramping and waving phases, respectively. Although the flexible joint waves vertically under an end load, the tracking errors do not contain obvious biases.

The second rows of Figure 12 and Figure 13 show accurate nonlinearity approximations, which validate the effectiveness of the NINA technique. The third rows of Figure 12 and Figure 13 indicate that the above control performances are achieved under relatively clean control inputs. It is noteworthy that the tracking errors under different end loads are nearly identical. This verifies the robustness of the proposed control method.

A classical RBFN-based adaptive control presented in [51] is compared with the proposed control method. On the load side, $\Lambda_{1}e_{\alpha }+{\dot{\alpha }}_{r}$, $\Lambda_{1}{\dot{e}}_{\alpha }+{\ddot{\alpha }}_{r}$, α, and $\dot{\alpha }$ are supplied to the input layer of RBFN. On the motor side, $\Lambda_{2}e_{\beta }+{\dot{\beta }}_{r}$, $\Lambda_{2}{\dot{e}}_{\beta }+{\ddot{\beta }}_{r}$, β, and $\dot{\beta }$ are supplied to the input layer of RBFN. Five neurons are set in hidden layers on the load side and the motor side. The control torques are obtained from the output layer of the RBFN. For more details, please refer to [51].

**Figure 14:**
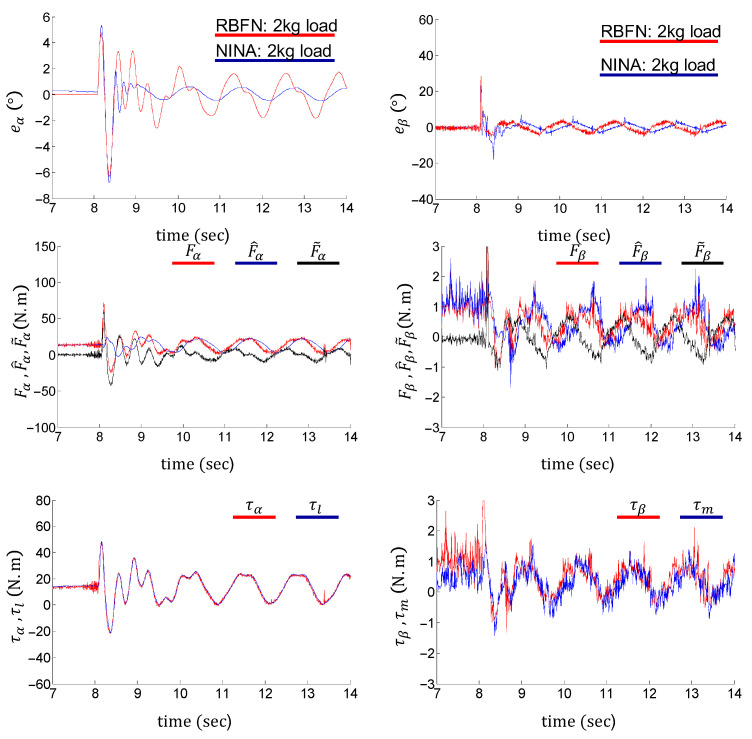
Performance of the RBFN adaptive controller under a 2 kg end load.

The performance of RBFN control is shown in Figure 14. In the switching phase, the tracking error of the proposed method converges faster than the RBFN-based method. In the swing phase, the load- and motor-side tracking errors of the RBFN control are bounded by $\left|e_{\alpha }\right|<$ 1.75° and $\left|e_{\beta }\right|<$ 3°, respectively. The tracking errors of the proposed NINA-based control method are bounded by $\left|e_{\alpha }\right|<$ 0.25° and $\left|e_{\beta }\right|<$ 3°. In addition, the nonlinearity approximation of the RBFN control (${\hat{F}}_{\alpha }$, ${\hat{F}}_{\beta }$) shows obvious lags behind their nominal values ($F_{\alpha }$, $F_{\beta }$), thereby resulting in relatively large estimation errors (${\tilde{F}}_{\alpha }$, ${\tilde{F}}_{\beta }$). The above comparison indicates that the proposed NINA-based adaptive control can realize better control performance than the RBFN-based adaptive control on the flexible joint system.

## 8. Conclusions

This study proposed a simplified adaptive control based on NINA for a class of nonlinear cascaded systems. The uniformity and ultimate stability of the proposed control were proven. The nonlinearities of each subsystem were approximated using the synthetic form of a steady component and an alternating component based only on local tracking errors. The proposed control method was validated through applications on the flexible joint system involving numerical simulations and experiments. The simulation results illustrated that the proposed method can achieve similar control accuracy as FT-SMC but uses milder control inputs. It was also indicated that the proposed method is insensitive to external loads and parametric perturbations. The proposed method was compared with an RBFN-based method. The experimental results demonstrated that the proposed method could achieve better control performance than an RBFN-based method.

Future work could be extended to flexible manipulators with variable stiffness. Future interests lie in two main areas: The first is optimizing the mapping process from the control input of the unactuated subsystem to the command layer of the actuated subsystem, which could improve the stability and noise level of the control system. The second is augmenting the adaptive law with a priori information on the system, to accelerate the convergence of the nonlinearity approximation.

## Figures and Tables

**Table 1:** Parameters of the two-link robot manipulator system.

Parameters	Value	Parameters	Value	Unit	Means
$m_{1}$	6.0	$m_{2}$	4.0	kg	mass of the link
$l_{1}$	0.6	$l_{2}$	0.4	m	length of the link
$d_{1}$	0.3	$d_{2}$	0.2	m	length of the mass center
$J_{1}$	$2.2\times 10^{-3}$	$J_{2}$	$6.4\times 10^{-4}$	$kg.m^{2}$	motor inertia
$f_{d1}$	0.088	$f_{d2}$	0.057	N.m/rad/s	motor damping
$k_{s1}$	120.0	$k_{s2}$	100.0	N.m/rad	joint stiffness
$k_{d1}$	$6.79\times 10^{-2}$	$k_{d2}$	$3.69\times 10^{-2}$	N.m/rad/s	elasticity damping

**Table 2:** Parameters of the proposed NINA-based adaptive control.

Parameters	Value	Parameters	Value
$\vartheta_{s1},\vartheta_{s2}$	0.3	$\vartheta_{s3},\vartheta_{s4}$	6
$K_{1}\sim K_{2}$	10	$K_{3}\sim K_{4}$	5
$A_{1}\sim A_{2}$	0.3	$A_{3}\sim A_{4}$	1
$B_{1}\sim B_{2}$	1000	$B_{3}\sim B_{4}$	1000
$\Lambda_{1}\sim \Lambda_{2}$	5	$\Lambda_{3}\sim \Lambda_{4}$	5

Note that subscripts 1 and 2 represent the motor-side control parameters of joints 1 and 2, respectively. Subscripts 3 and 4 represent the load-side control parameters of joints 1 and 2, respectively.

**Table 3:** Tracking performance of the three methods.

		SSP		CTP		
		Joint 1	Joint 2	Joint 1	Joint 2	Average
Link Side	NINA	−0.07°~0.08°	−0.03°~0.03°	1.75 s	1.78 s	1.77 s
	FT-SMC	−0.05°~0.09°	−0.07°~0.10°	1.88 s	1.24 s	1.56 s
	RBFN	−0.17°~0.16°	0.20°~0.20°	2.73 s	1.74 s	2.24 s
Motor Side	NINA	−0.11°~0.12°	−0.13°~0.13°	0.50 s	0.20 s	0.35 s
	FT-SMC	−0.21°~0.09°	−0.07°~0.10°	0.82 s	0.90 s	0.86 s
	RBFN	−0.29°~0.17°	−0.18°~0.19°	1.24 s	1.09 s	1.17 s

**Table 4:** Nonlinearity estimation performance of the three methods.

		SSE		CTE		
		Joint 1	Joint 2	Joint 1	Joint 2	Average
Link Side	NINA	−0.078~0.16 Nm	−0.12~0.11 Nm	0.7 s	0.4 s	0.55 s
	FT-SMC	−0.28~0.50 Nm	−0.19~019 Nm	1.9 s	1.7 s	1.8 s
	RBFN	−1.60~1.60 Nm	−0.8~0.8 Nm	2.76 s	1.9 s	2.33 s
Motor Side	NINA	−0.27~0.52 Nm	−0.064~0.006 Nm	0.24 s	0.19 s	0.22 s
	FT-SMC	−1.05~0.75 Nm	−0.14~0.14 Nm	0.9 s	1.3 s	1.10 s
	RBFN	−2.14~0.55 Nm	−0.66~064 Nm	1.2 s	1.4 s	1.30 s

**Table 5:** Parameters of the flexible joint system.

Measured Values of Mechanical Parameters			Control Parameters			
$J_{\alpha }$	1.090, under 2 kg end load	$kg.m^{2}$	$\vartheta_{s1}$	1.125000	$\vartheta_{s2}$	0.030000
	1.840, under 4 kg end load	$kg.m^{2}$	$K_{1}$	0.135000	$K_{2}$	0.010000
$g_{\alpha }$	15.12, under 2 kg end load	N.m	$\Lambda_{1}$	26.50000	$\Lambda_{2}$	200.0000
	24.08, under 4 kg end load	N.m	$A_{1}$	0.000180	$A_{2}$	0.000001
$J_{\beta }$	4.65$\times 10^{-4}$	$kg.m^{2}$	$\lambda_{1}$	125000.0	$\lambda_{2}$	100000.0
$k_{s}$	927.0	N.m/rad				
$k_{d}$	1.54	N.m/rad/sec				
η	50.0	—				

Note that subscripts α and β represent the load and motor sides of the flexible-joint platform, respectively.

## Data Availability

Data are contained within the article.
